# Comparative Analysis of Pharmacodynamics in the C3HeB/FeJ Mouse Tuberculosis Model for DprE1 Inhibitors TBA-7371, PBTZ169, and OPC-167832

**DOI:** 10.1128/AAC.00583-21

**Published:** 2021-10-18

**Authors:** Gregory T. Robertson, Michelle E. Ramey, Lisa M. Massoudi, Claire L. Carter, Matt Zimmerman, Firat Kaya, Barbara G. Graham, Veronica Gruppo, Courtney Hastings, Lisa K. Woolhiser, Dashick W. L. Scott, Bryce C. Asay, Franceen Eshun-Wilson, Ekaterina Maidj, Brendan K. Podell, Joshua J. Vásquez, Mike A. Lyons, Véronique Dartois, Anne J. Lenaerts

**Affiliations:** a Mycobacteria Research Laboratories, Department of Microbiology, Immunology and Pathology, Colorado State University, Fort Collins, Colorado, USA; b Center for Discovery and Innovation and Hackensack School of Medicine, Hackensack Meridian Health, Nutley, New Jersey, USA; c Division of Experimental Medicine & Division of Pulmonary and Critical Care Medicine, University of California, San Francisco, California, USA; d Consortium for Applied Microbial Metrics

**Keywords:** tuberculosis, murine models, DprE1 inhibitors, C3HeB/FeJ

## Abstract

Multiple drug discovery initiatives for tuberculosis are currently ongoing to identify and develop new potent drugs with novel targets in order to shorten treatment duration. One of the drug classes with a new mode of action is DprE1 inhibitors targeting an essential process in cell wall synthesis of Mycobacterium tuberculosis. In this investigation, three DprE1 inhibitors currently in clinical trials, TBA-7371, PBTZ169, and OPC-167832, were evaluated side-by-side as single agents in the C3HeB/FeJ mouse model presenting with caseous necrotic pulmonary lesions upon tuberculosis infection. The goal was to confirm the efficacy of the DprE1 inhibitors in a mouse tuberculosis model with advanced pulmonary pathology and perform comprehensive analysis of plasma, lung, and lesion-centric drug levels to establish pharmacokinetic-pharmacodynamic (PK-PD) parameters predicting efficacy at the site of infection. Results showed significant efficacy for all three DprE1 inhibitors in the C3HeB/FeJ mouse model after 2 months of treatment. Superior efficacy was observed for OPC-167832 even at low-dose levels, which can be attributed to its low MIC, favorable distribution, and sustained retention above the MIC throughout the dosing interval in caseous necrotic lesions, where the majority of bacteria reside in C3HeB/FeJ mice. These results support further progression of the three drug candidates through clinical development for tuberculosis treatment.

## INTRODUCTION

Tuberculosis (TB), an infectious disease caused by Mycobacterium tuberculosis, constitutes a serious global health threat (1, 2). In 2019 alone, there were an estimated 10 million new diagnosed TB cases worldwide, with TB deaths reaching more than 1.5 million, including 250,000 HIV-positive individuals, thereby still making TB the leading cause of death from an infectious disease in 2019 (2). Moreover, the occurrence of multidrug-resistant (MDR) TB with more than 500,000 cases recorded worldwide, the concurrent epidemics of HIV-TB and SARS-CoV-2 coinfection with TB, the lack of adequate health care infrastructure, and the absence of an effective TB vaccine further fuel the disease burden (1, 3, 4). Despite the fact that most TB cases are potentially curable, the prolonged nature of treatment regimens, especially for MDR-TB, as well as the potential toxicity of second-line TB agents further complicate the ability to eradicate the disease. Although three new agents, bedaquiline (BDQ), pretomanid (PMD), and delamanid (DMD) (5), were recently approved for the treatment of MDR-TB, additional new drugs with a novel mechanism of action (MOA) are still urgently needed to shorten treatment and improve treatment safety profiles (6).

The current TB drug development pipeline shows extensive research activity in the drug discovery stage (www.newtbdrugs.org); however, a significant gap remains in the late preclinical and phase I clinical stages to balance attrition during the later phases and to secure continuity of clinical candidates for new regimen development (7). To combat this problem, scientists around the world have formed consortia using a variety of approaches directed to develop TB drugs with a new mode of action or new target (8). The decaprenylphosphoryl-beta-d-ribose oxidase (DprE1) enzyme is a novel drug target in M. tuberculosis that is vulnerable due to its essentiality in mycobacteria and its location in the bacterial cell wall (9). The DprE1 enzyme, together with its partner enzyme DprE2, catalyzes the conversion of decaprenylphosphoryl-β-d-ribose (DPR) to its arabinose counterpart, decaprenylphosphoryl-β-d-arabinose (DPA), which is the only donor of arabinose sugars that are essential for cell wall biosynthesis for mycobacteria and *Corynebacterineae* species (10, 11). The periplasmic location of its active site in the cell wall accounts for the fact that DprE1 has been identified in recent high-throughput screens (HTS) as the target of numerous compounds with structurally distinct pharmacophores (12), earlier termed promiscuous targets (13). To date, more than 11 different structural scaffolds have been reported, exhibiting high binding affinity to the enzyme with various physicochemical properties that affect their activity *in vitro* and *in vivo* and PK/PD profiles. The DprE1 enzyme therefore appears to be a highly druggable target, as it is inhibited by different chemical scaffolds (reviewed in reference 12). Most DprE1 inhibitor series were discovered during phenotypic or high-throughput screens of compound libraries or databases; lead compounds were identified and further chemically optimized. Most early compound series were covalent inhibitors, and more recently multiple chemical scaffolds were discovered that inhibit the target noncovalently (12), with some showing *in vivo* efficacy. At least four drug candidates targeting the DprE1 enzyme are currently in clinical trials undergoing evaluation for safety and effectiveness. These are BTZ043, pursued by the University of Munich and Hans Knöll Institute, macozinone (MCZ; PBTZ169), by the Innovative Medicines for Tuberculosis (iM4TB) consortium and Nearmedic Plus LLC, TBA-7371, by the TB Alliance, and OPC-167832, by Otsuka.

The benzothiazinones (BTZs) were discovered in 2009, with the lead compound BTZ043 showing high potency (MIC of 1 ng/ml) against M. tuberculosis H37Rv, activity against MDR and extensively resistant M. tuberculosis isolates, and *in vivo* efficacy (9). The BTZs and other reported nitroaromatic DprE1 inhibitors are covalent suicide inhibitors of DprE1. Piperazine-containing BTZ (PBTZ) derivatives were then designed to improve the pharmacological properties of the molecule (14), which led to the optimized lead compound, PBTZ169. PBTZ169 is 10 times less cytotoxic *in vitro* than BTZ043, with both compounds having a selectivity index of >10,000. The MIC of PBTZ169 was 3-fold lower versus BTZ043, attributed to the fact PBTZ169 inhibits the DprE1 target more efficiently. Both compounds show very high plasma protein binding of >99.8%, with increased solubility for PBTZ169 at low pH versus BTZ043, which could account for more rapid absorption. PBTZ169 shows increased efficacy in a chronic BALB/c mouse model, showing an additional 0.5-log_10_ CFU reduction in lungs and an additional 1-log_10_ CFU in spleens versus BTZ043, resulting in a 1-log_10_ CFU reduction in lungs versus untreated controls after 4 weeks of treatment (14). Differences in PK are unlikely contributors to the *in vivo* benefit of PBTZ169 over BTZ043, as both compounds display similar PK properties.

A second DprE1 inhibitor, TBA-7371, was developed in collaboration between Astra Zeneca and the TB Alliance (15–17). TBA-7371 is a derivative of 1,4-azaindoles, synthesized through scaffold morphing of an imidazopyridine compound. It is a noncovalent inhibitor of M. tuberculosis DprE1 with a MIC of 0.64 μg/ml and a similar minimum bactericidal concentration (MBC) (16). TBA-7371 shows a good *in vitro* safety profile without cytotoxicity up to 100 μM. Modification of the hit azaindole compound focused on improvement of solubility and the rapid *in vivo* clearance rate, which led to the clinical candidate TBA-7371. The compound shows good efficacy in an acute BALB/c mouse model, showing more than 2-log_10_ CFU reduction in lungs dosed at 100 mg/kg of body weight once a day (QD) compared to untreated controls. In a chronic BALB/c mouse model, TBA-7371 resulted in a 1.5-log_10_ CFU reduction in lungs at 100 mg/kg QD.

Another phenotypic screening effort around the carbostyril core, known to have good ADME (absorption-distribution-metabolism-excretion) and toxicity properties, resulted in OPC-167832 (18). The MIC of OPC-167832 is low, ranging from 0.24 to 2 ng/ml, with bactericidal activity against both replicating and intracellular bacteria and a low frequency of resistance of <1.9 × 10^−7^. Both whole-genome sequencing of resistant isolates and enzymatic assays validated DprE1 as the target. In a chronic ICR mouse model, infected intratracheally with M. tuberculosis Kurono, OPC-167832 showed bactericidal activity at 0.625 mg/kg and resulted in more than a 1.5-log_10_ CFU reduction in lungs versus the start of treatment when administered for 4 weeks at 5 mg/kg.

Murine models are employed in tuberculosis research due to their small size and low cost. In addition, their physiology and genetics are well understood. Standard laboratory mouse strains, such as C57BL/6 and BALB/c, have been used most widely in TB drug development (19, 20). However, these mouse strains show some limitations by solely developing a single lesion type after M. tuberculosis infection and therefore lack the lesion heterogeneity seen in the lungs of TB patients (19, 20). The C3HeB/FeJ mouse infection model presents with a heterogeneity of pulmonary TB lesion types more reflective of human disease (21–31) and is currently employed primarily in late-stage TB drug and regimen development (26, 32–35). This mouse model was first described for tuberculosis by Kramnik et al. and is commonly referred to as the “Kramnik mouse model” (36). The lung pathology of infected C3HeB/FeJ mice presents with three distinct lesion types: collagen-encapsulated caseous necrotic lesions (earlier classified as type I lesions), fulminant neutrophilic alveolitis (type II lesions), and cellular nonnecrotizing lesions (type III lesions), with only the latter lesion type being present in BALB/c mice (23). The predominant type I lesions are well-organized, caseous necrotic granulomas in C3HeB/FeJ lungs that contain a central core composed of primarily neutrophilic debris surrounded by a cellular layer, which is bordered by a collagen rim with interstitial macrophages admixed within the rim (23, 32). The cellular layer is composed of a foamy macrophage cell cuff immediately adjacent to the collagen rim (here defined as the rim), with a layer of live as well as dead, but intact, neutrophils delineating the caseum of acellular debris (23). Type I lesions contain the majority of the bacteria (approximately 10^6^ to 10^7^ per lesion), which are located extracellularly in the lesion core (here defined as caseum) or intracellularly in the foamy macrophage and neutrophil layers (here defined as the cellular-caseum interface) (23). Infected mice also present with smaller cellular type III lesions throughout the lung, which only contain a limited bacterial burden (approximately 10^2^ to 10^4^ per lesion). Due to the substantially different microenvironments in type I lesions, which vary in oxygen tension, pH, carbon source, and cellular composition, multiple bacterial phenotypes are present. A profound impact has been observed on drug distribution and drug activity against the various bacterial populations in caseum, both of which are known to affect the *in vivo* efficacy of certain drugs (27, 32, 37). The differential treatment response is not unique to the C3HeB/FeJ mouse model and has also been observed for animal models with similar pulmonary pathologies, such as the NHP, marmoset, and rabbit models (reviewed in reference 27).

For the work presented here, a comprehensive analysis was made to link drug efficacy and exposure for the three DprE1 inhibitors at the locations in lung lesions where the majority of bacteria reside. The goal was to confirm the efficacy of the DprE1 inhibitors in a murine tuberculosis model with advanced pulmonary pathology and perform thorough analysis of plasma, lung, and lesion-centric PK/PD by testing three DprE1 inhibitors side-by-side in a single animal model.

## RESULTS

### *In vitro* activity of DprE1 inhibitors.

**FIG 1:**
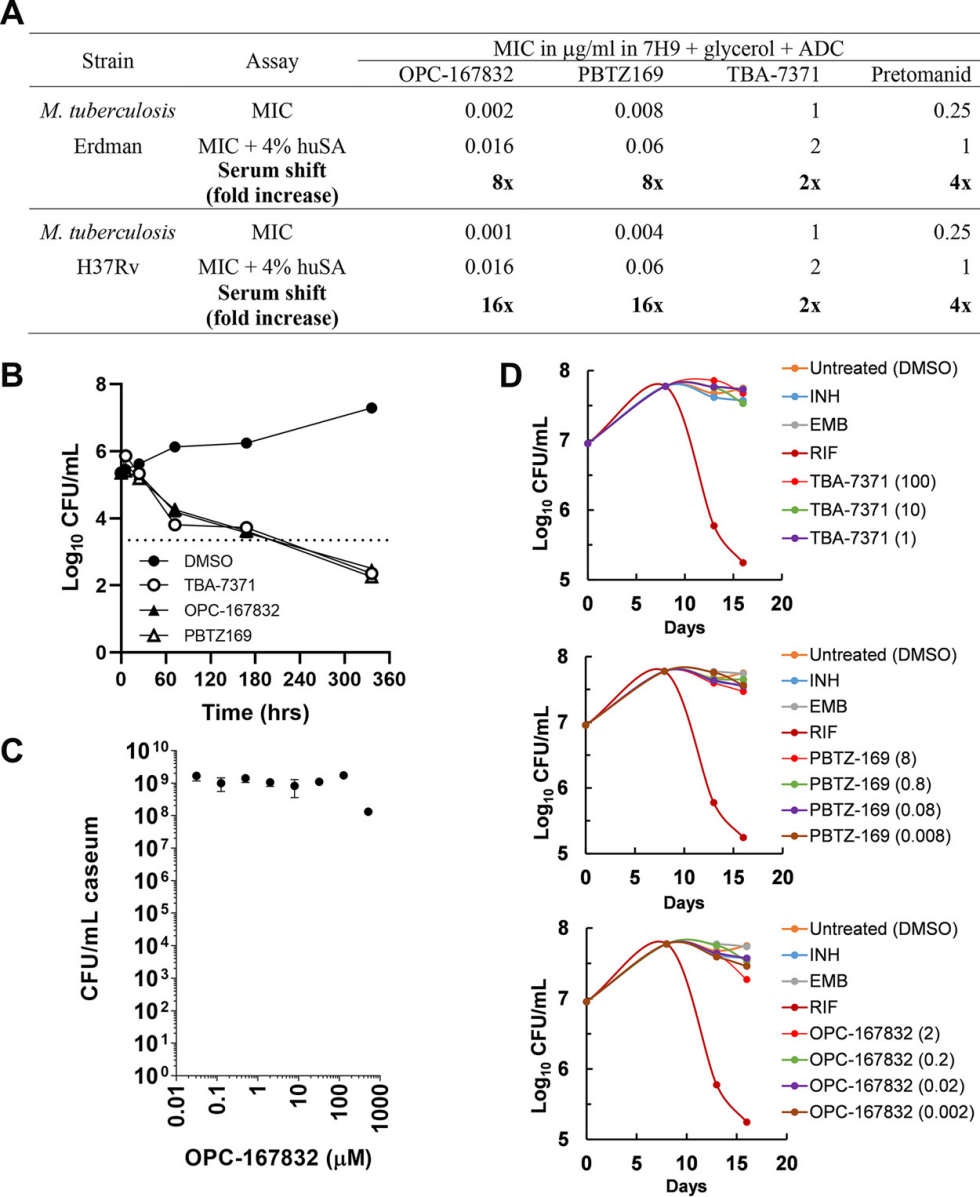
*In vitro* antimicrobial activity of DprE1 inhibitors OPC-167832, PBTZ169, and TBA-7371 against actively replicating M. tuberculosis and nonreplicating bacteria. (A) MIC values in presence and absence of 4% human serum albumin (huSA). Pretomanid was used as a positive control. (B) *In vitro* kill kinetics at 4× the MIC of TBA-7371, OPC-167832, and PBTZ-169 compared to the DMSO vehicle negative control using M. tuberculosis Erdman under replicating conditions. The dashed line indicates the 2-log kill or 99% CFU reduction of the starting inoculum. (C) Bactericidal activity of OPC-167832 using an *in vitro* assay with rabbit caseum. Data are expressed as average log_10_ CFU numbers per milliliter of homogenized caseum from three replicates. Standard deviations are indicated by error bars. (D) *In vitro* kill kinetics by log_10_ CFU determination after drug treatment (in μg/ml) against M. tuberculosis grown under oxygen depletion. Isoniazid (INH) and ethambutol (EMB) were used as negative controls. They are known to lack activity in this assay. Rifampin (RIF) was used as a positive control.

The *in vitro* activity of OPC-167832, PBTZ169, and TBA-7371 was evaluated against actively replicating M. tuberculosis H37Rv and Erdman strains as well as against nonreplicating bacteria grown under hypoxic conditions and in caseum. In a broth microdilution assay, the MIC values for OPC-167832, PBTZ169, and TBA-7371 were 0.002, 0.008, and 1 μg/ml, respectively (Fig. 1A). In the presence of 4% human serum albumin (huSA), there was an 8-fold shift in MIC observed for both OPC-167832 and PBTZ169 to 0.016 and 0.06 μg/ml, respectively, indicating ∼88% protein binding by this assay. For TBA-7371, there was a 2-fold shift in MIC observed in the presence of 4% huSA, which is within the standard deviation of the assay. The reported plasma protein binding for PBTZ169 and TBA-7371 were 99.84% (personal communication from the supplier) and 79% (15, 16), respectively. *In vitro* kill kinetics of the three DprE1 inhibitors were determined against M. tuberculosis Erdman under replicating conditions at 4× their respective MIC. Results showed similar kill kinetics for the three inhibitors, as determined by number of CFU/ml over 14 days by serial dilution and culture on 7H11 agar plates with 0.4% activated charcoal (Fig. 1B).

All three DprE1 inhibitors, OPC-167832, PBTZ169, and TBA-7371, were evaluated against M. tuberculosis grown under gradual oxygen depletion in the rapid anaerobic dormancy (RAD) model (38) to confirm limited or lack of activity against nonreplicating bacteria, as is expected for cell wall synthesis inhibitors. Concentrations for all three compounds, ranging from 1× to 1,000× MIC, were chosen based on drug levels measured in pulmonary tissues in this report. Results showed no *in vitro* activity against nonreplicating bacteria under hypoxic conditions for both PBTZ169 and TBA-7371, whereas OPC-167832 showed a marginal effect only at the highest concentration of 1,000× MIC (Fig. 1D). As a follow-up study, OPC-167832 was also evaluated in an MBC caseum assay against nonreplicating bacteria using dissected caseum from M. tuberculosis-infected rabbits. Similar to the results obtained in the RAD assay, OPC-167832 showed a statistically significant reduction of 1 log from treatment start only at the highest concentration tested (1 mg/ml) (*P* = 0.0005), with an MBC_90_ of 500 μg/ml, which is substantially above the found drug levels measured in mouse lungs (Fig. 1C).

### Efficacy of DprE1 inhibitors in lungs of C3HeB/FeJ mice.

The efficacy of DprE1 inhibitors was evaluated in M. tuberculosis Erdman-infected C3HeB/FeJ mice after 4 and 8 weeks of treatment with TBA-7371 (at 50, 100, and 200 mg/kg, dosed twice a day [BID]), PBTZ169 (at 25, 50, 100 mg/kg, dosed QD), and OPC-167832 (at 1.25, 5, 20 mg/kg, dosed QD). Treatment was initiated 8 weeks after aerosol infection to allow for sufficient time for mice to develop pulmonary caseous necrotic lesions. The dose range was centered on the reported individual MED_99_ of the compounds (MED_99_ is the minimal effective dose resulting in a 2-log reduction in the bacterial load in lungs versus untreated controls after treatment, generally in an acute M. tuberculosis infection mouse model). From published or reported mouse infection studies, the MED_99_ for TBA-7371 was 100 mg/kg QD (15), the MED_99_ for PBTZ169 was 50 mg/kg QD, and the MED_99_ for OPC-167832 was 5 mg/kg QD (18). The two additional dose levels tested were 0.5- and 2-fold the MED_99_ for both TBA-7371 and PBTZ169 and 0.25- and 4-fold the MED_99_ for OPC-167832. Both PBTZ169 and OPC-167832 were administered QD, whereas TBA-7371 was administered BID based on rapid drug clearance in mice compared to human, which was derived from available clinical pharmacological data (personal communication from the supplier). As expected, the lungs of C3HeB/FeJ mice at the start of treatment presented with the typical spectrum of lesion types, with most mice showing the characteristic caseous necrotic lesions. The bacterial load in lungs for the untreated, infected control C3HeB/FeJ mice showed a slight increase over time, as expected (6.73 log_10_ CFU ± 0.21 standard errors of the means [SEM] at 8 weeks after aerosol, 7.59 log_10_ CFU ± 0.32 SEM at 12 weeks after aerosol, and 8 log_10_ CFU ± 0.16 SEM at 16 weeks after aerosol).

**FIG 2:**
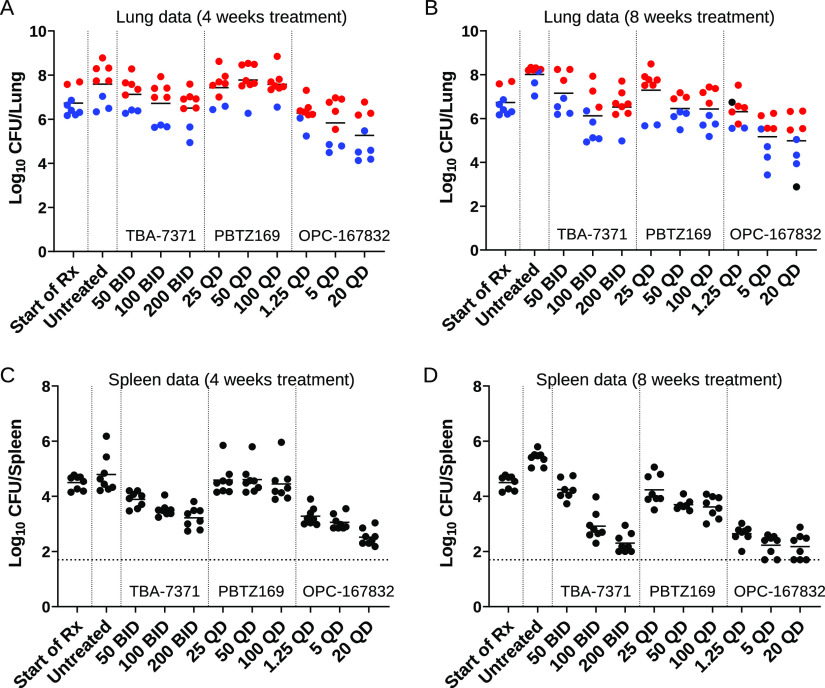
Efficacy of DprE1 inhibitors in C3HeB/FeJ mice after 4 and 8 weeks of treatment with TBA-7371, PBTZ169, and OPC-167832. Eight weeks following a low-dose aerosol infection, mice were treated 5/7 days (Monday to Friday) via oral gavage at the doses (mg/kg) and dosing frequencies indicated on the *x* axis. Plots represent log_10_ CFU determinations in lungs (A and B) and spleens (C and D) of individual C3HeB/FeJ mice after 4 (A and C) and 8 weeks (B and D) of treatment. Pretreatment controls (start of Rx) represent untreated mice infected for 8 weeks. Lung lesion analysis was performed on blinded digital photographs taken at gross necropsy. Results in red represent mice that showed pronounced type I (caseous necrotic) lesions at gross necropsy. Results in blue represent mice without pronounced type I lesions at gross necropsy and only small type I and/or type III (inflammatory) lesions. Results in black represent mice with possible type I lesions, but these were unclear on the photograph. The lower limit of CFU detection in spleens was 1.7 logs (indicated with a horizontal dashed line).

In lungs after 4 weeks of treatment, only the OPC-167832 20-mg/kg treatment group showed statistically significant efficacy versus the pretreatment control group (*P = *0.04 by Dunnett’s test; Fig. 2A). Compared to the untreated control group after the 4-week treatment period, all three OPC-167832 treatment groups showed a statistically significant reduction in lung bacterial load (*P = *0.002 to 0.03 by Dunnett’s test). The effects of TBA-7371 and PBTZ169 on lung CFU compared to pretreatment control and untreated controls were not statistically significant (*P > *0.05). The calculated rate of kill (*k*; Δlog_10_ CFU/day) in lungs was shown to be highest and also dose dependent for OPC-167832 during the first 4 weeks of treatment (see Table S1A in the supplemental material).

After 8 weeks of treatment, results in lungs showed similar efficacy for the PBTZ169 and TBA-7371 treatment groups at the doses tested (Fig. 2B). Maximal efficacy results were achieved at the earlier established MED_99_ for both PBTZ169 (at 50 mg/kg, QD) and TBA-7371 (at 100 mg/kg, BID), which can be defined as the LME (lowest dose with the maximum effect) for these compounds in the C3HeB/FeJ mouse model. OPC-167832 showed superior efficacy for the middle- and high-dose groups compared to PBTZ169 and TBA-7371 (Fig. 2B). Maximal efficacy was achieved at the established MED_99_ for OPC-167832 (5 mg/kg, QD), which is also the LME for this compound in C3HeB/FeJ mice. When measuring the kill rates after 8 weeks, several additional observations could be made regarding the different kill kinetics by the three DprE1 inhibitors (Table S1A). First, the kill rate for OPC-167832 during the second month of treatment was substantially lower than that during the first 4 weeks, indicating that OPC-167832 efficacy was superior early on. In addition, the highest OPC-167832 dose group was less effective than the middle-dose group (0.01/day versus 0.024/day, respectively), indicating that an increase in dose level is not necessarily beneficial in the second month of treatment. In contrast, a negative rate of kill was observed for the first month of PBTZ169 treatment, followed by a high kill rate during the second month of treatment (0.047/day and 0.041/day at 50 and 100 mg/kg, respectively). The PBTZ169 kill rate during the second month of treatment was as high as OPC-167832 at 20 mg/kg during the first month of treatment. A more modest kill rate was observed for TBA-7371, which was more consistent over time, especially for the 100 mg/kg BID group (Table S1A).

Statistical analysis of the 8-week treatment CFU numbers in lungs showed only statistically significant efficacy for OPC-167832 (at 5 and 20 mg/kg) versus the pretreatment control group (*P = *0.01 and 0.03, respectively, by Dunnett’s test). Compared to the untreated control group after the treatment period, all three OPC-167832 treatment groups as well as TBA-7371 (medium, high dose) and PBTZ169 (medium, high dose) showed statistical significant reduction in the lung bacterial load (*P < *0.05 by Dunnett’s test). When analyzing the efficacy of the various drug treatment groups in lungs in pairwise comparisons, the OPC-167832 groups were the only ones showing statistical superiority versus the other treatment groups (Table S2A and B). At 4 weeks, OPC-167832 (at the high dose) was shown to be more efficacious than TBA-7371 (low, medium dose) and PBTZ169 (low, medium, high); OPC-167832 (medium) was significantly more effective than PBTZ169 (low, medium, high); and OPC-167832 (low) was more effective than PBTZ169 (medium) (Table S2A). At 8 weeks, OPC-167832 (high) was more efficacious than TBA-7371 (low, high) and PBTZ169 (low). OPC-167832 (medium) was significantly better than TBA-7371 (low) and PBTZ169 (low) (Table S2C).

Using a three-way analysis of variance (ANOVA) followed by a Tukey test, the bacterial loads in tissues were compared for differences based on the drug treatments (OPC-167832, TBA-7371, and PBTZ169) and the dosage tested (low, medium, or high) at two time points (4 weeks and 8 weeks). Although there were significant differences in efficacy between drugs, drug dose did not have a clear effect in terms of statistical significance on the bacterial numbers found in lung tissue (data not shown). Significant differences were only seen between the two treatment groups with the lowest bacterial numbers by CFU (OPC-167832 medium, high) versus the two treatment groups with the highest bacterial numbers (PBTZ169 low, TBA-7371 low) (*P < *0.05 by Tukey test).

### Caseous necrosis impacts efficacy of DprE1 inhibitors in lungs of C3HeB/FeJ mice.

Treatment of C3HeB/FeJ mice with single agents is often associated with distinct treatment responses based on histopathology, whereby two distinct populations of mice emerge, defined as responsive in mice with less severe pathology and less responsive in animals with severe pulmonary pathology, as described in previous studies (32). The most effective compounds will be effective against all lesion types; therefore, studying the treatment response based on lesion types for a new compound can provide valuable additional information. Visual inspection of lungs was performed on blinded digital images taken at gross necropsy to distinguish mice with and without visible large type I lesions to determine if treatment response could be directly linked to the heterogeneous histopathology observed in lungs of individual animals. Results showed for animals with the most advanced necrotic lesions a clear reduction in efficacy for the three DprE1 inhibitors (Fig. 2A and B, red). The animals that presented with small type I lesions (not visible by eye at gross necropsy) as well as type III lesions were more responsive to treatment (Fig. 2A and B, blue).

To determine whether the three DprE1 inhibitors exhibited lesion-specific kill kinetics, kill rates were determined in mouse populations with either predominantly large type I or type III lesions. The latter also included small type I lesions that are not or are less visible at gross necropsy. For this purpose, visible inspection of lungs was performed on blinded digital images taken at gross necropsy (as described above) to allocate mice either to the type I (TI) or type III (TIII) lesion groups. This analysis revealed higher kill rates for TBA-7371 and OPC-167832 in mice with cellular TIII lesions than those in mice with large TI lesions in the first month of treatment (Table S1B). During the second month of treatment, the PBTZ169 kill rate was also higher in cellular type III lesions than type I lesions, with the exception of the intermediate dose, where kill rates were higher in mice with type I lesions. In contrast, OPC-167832 exhibited a higher kill rate in large caseous necrotic type I lesions than in type III lesions at both 5 and 20 mg/kg during the second month of treatment (Table S1B). This observation should be considered with some degree of caution due to higher uncertainty owing to limited numbers of data points per group.

### Efficacy of DprE1 inhibitors in spleens of C3HeB/FeJ mice.

In spleens, the bacterial load for the untreated, infected control C3HeB/FeJ mice showed a slight increase over time, as expected (4.50 log_10_ CFU ± 0.09 SEM at 8 weeks after aerosol, 4.79 log_10_ CFU ± 0.24 SEM at the 12 weeks, and 5.39 log_10_ CFU ± 0.26 SEM at 16 weeks). As previously described, the spleens showed solely cellular lesions, as observed in the classical BALB/c chronic TB mouse infection model (23).

After 4 weeks, PBTZ169 showed no statistically significant CFU reduction in spleen at any dose, whereas both TBA-7371 and OPC-167832 showed dose-dependent CFU reduction (Fig. 2C). After 4 weeks, all OPC-167832 and TBA-7371 treatment groups showed statistically significant CFU reduction compared to untreated controls (*P < *0.05), and all three OPC-167832 and two of three TBA-7371 (i.e., 100 and 200 mg/kg) treatment groups showed statistically significant reduction in spleen burden compared to untreated controls (*P < *0.05).

After 8 weeks, results in spleens showed again similar efficacy for both the OPC-167832 and TBA-7371 treatment groups at the medium and high doses tested, respectively (Fig. 2D). Maximal efficacy was achieved at the earlier established MED_99_ for OPC-167832 (at 5 mg/kg, QD), whereas for TBA-7371 maximal efficacy in spleens was not yet achieved (≥200 mg/kg, BID).

Analysis of kill rates in spleens further revealed markedly different kill kinetics of the three DprE1 inhibitors (see Table S1C). First, the kill rate for OPC-167832 after 8 weeks was lower than that after 4 weeks, as seen in lungs. Although the difference was less pronounced than that in lungs, the kill rate of the high OPC-167832 dose group appeared slightly lower than that of the middle dose group in spleens (0.012/day versus 0.03/day), suggesting that a higher dose level is not beneficial in the second month of treatment. As observed in lungs, PBTZ169 exhibited a negative kill rate during the first month, followed by a higher kill rate in the second month (0.032/day and 0.030/day at 50 and 100 mg/kg, respectively). A consistent kill rate was observed throughout the 8-week treatment duration for TBA-7371, resulting in a clear dose response at all doses tested (see Table S1C).

Statistical analysis of the spleen data after 4 weeks of treatment showed that OPC-167832 (high) was more efficacious than TBA-7371 and PBTZ169 (at all dose levels), and OPC-167832 (medium) was significantly better than PBTZ169 (all dose levels) and TBA-7371 (low) (Table S2B). At 8 weeks of treatment, results showed significant efficacy for all treatment groups versus the pretreatment control group, except for TBA-7371 (100 mg/kg) and PBTZ169 (25 mg/kg) (data not shown). Compared to the untreated control group after the treatment period, all treatment groups showed statistically significant reductions in bacterial load in spleens (*P < *0.05). When comparing the efficacy of the various drug treatment groups in spleens in pairwise comparisons (two-way ANOVA, *P < *0.05), the OPC-167832 groups were the only ones showing statistical superiority versus the other treatment groups (*P < *0.05) (Tables S2B and D). OPC-167832 (high) showed efficacy similar to that of TBA-7371 (high), whereas OPC-167832 (medium) showed slightly better efficacy than TBA-7371 (medium). OPC-167832 (low) was significantly better than all three dose levels of PBTZ169. At 8 weeks, PBTZ169 showed statistical efficacy against the untreated controls (*P = *0.0025 to <0.0001 by Dunnett’s test) but was less effective at the medium and high dose in spleens than the other two DprE1 inhibitors (Fig. 2D).

### Effects of DprE1 inhibitors on pulmonary histopathology of C3HeB/FeJ mice.

**FIG 3:**
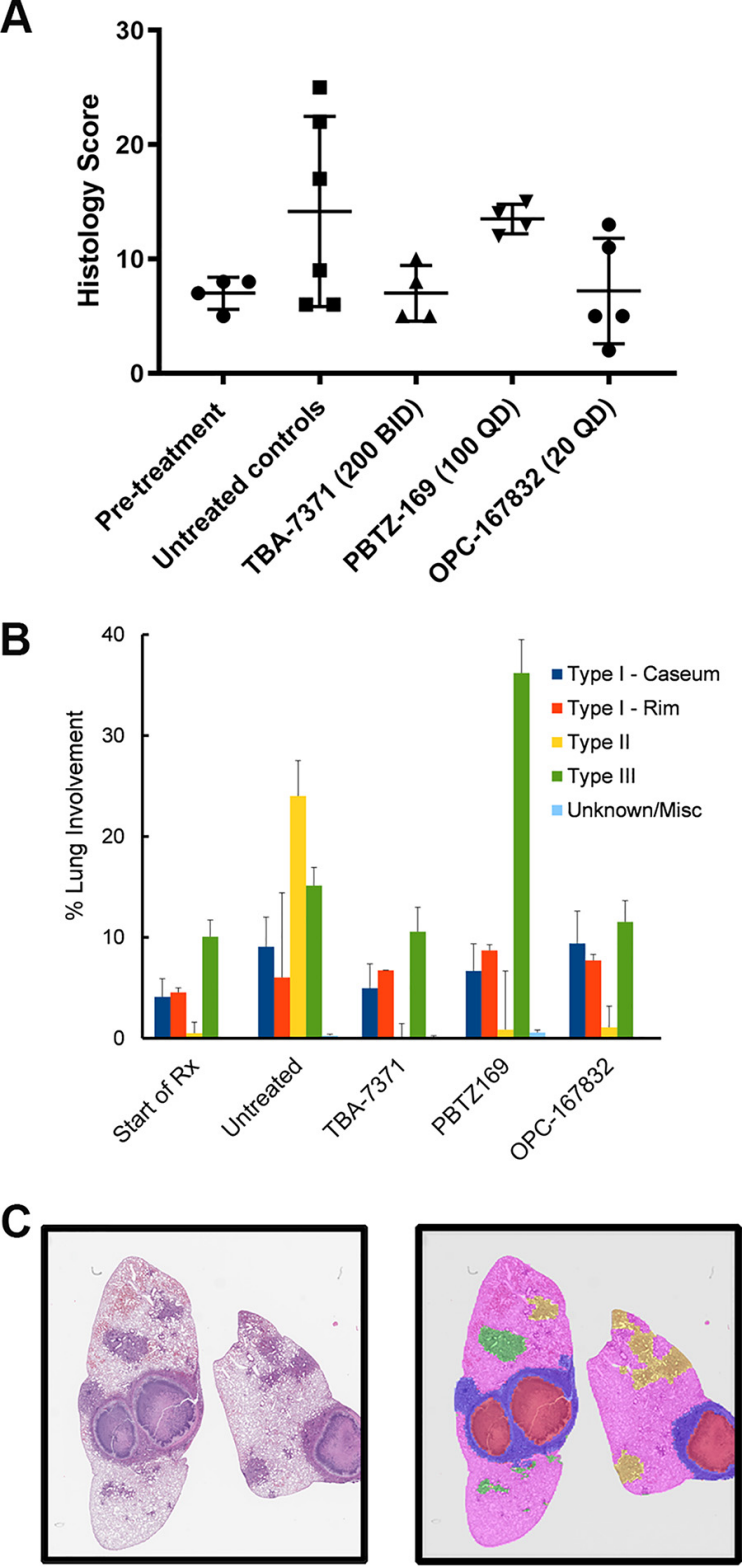
Histopathology analysis of pulmonary sections of C3HeB/FeJ mice at the start of treatment (start of Rx) and in untreated controls (untreated) or after 8 weeks of treatment with TBA-7371 (at 200 mg/kg, BID), PBTZ169 (at 100 mg/kg, QD), and OPC-167832 (at 20 mg/kg, QD). (A) Blinded histopathology analysis by a board-certified pathologist using a semiquantitative method based on histology scores (56). (B) Quantitative analysis plus the SEM using pathologist-assistive software called LIRA (lesion image recognition and analysis [21]) developed for pulmonary pathology of M. tuberculosis-infected C3HeB/FeJ mice, employing convolutional neural networks trained using a machine learning approach, and (C) visual representation of the results generated by LIRA for digital images of pulmonary lesions of drug-treated C3HeB/FeJ mice. The various lesion classifications are displayed in a color overlay depicting type I core (red), type I rim (blue), type II (green), type III (yellow), healthy tissue (pink), miscellaneous (purple), and empty slide (no color). A hematoxylin- and eosin-stained image is included for reference (left).

Histopathology analysis was performed on microscopic slides of fixed lung sections after 2 months of treatment using two complementary approaches. First, semiquantitative and qualitative pathology analysis was performed on blinded samples by a board-certified pathologist, using scoring of lung involvement per lesion type and description of pathology features for every slide. Results for the manual analysis showed that all three DprE1 inhibitors were able to halt progression of lung pathology from treatment start, with the best results obtained for TBA-7371 and OPC-167832 (Fig. 3A). Qualitative analysis of the PBTZ169 treatment group showed severe pneumonic disease consistent with type III cellular lesions, with the area of type III lesions showing more advanced lung involvement than untreated controls at the end of the treatment period.

Second, digital scans of hematoxylin and eosin (H&E)-stained tissue sections were evaluated using a new pathologist-assistive software called LIRA (lesion image recognition and analysis [21]), designed and developed for digital image analysis of pulmonary pathology in M. tuberculosis-infected C3HeB/FeJ mice. The LIRA results showed that all three DprE1 inhibitors were able to halt the progression of the lung pathology from treatment start by preventing the development of destructive type II neutrophilic lesions (which account for 25% of the total lung area in the untreated controls after 8 weeks) (Fig. 3B). In addition, LIRA showed a high incidence of type III cellular lesions in PBTZ169-treated mice, resulting in more than 35% lung involvement, which was more than 2-fold that observed for the untreated control group (Fig. 3B). A representative lung image pre- and post-LIRA classification is shown for reference (Fig. 3C).

### RS ratio indicates slow bacterial replication in caseous necrotic lesions using *in situ* hybridization.

A novel molecular biomarker was utilized to distinguish the multiple bacterial populations present in pulmonary necrotic lesions of infected C3HeB/FeJ mice based on replication status (39). In M. tuberculosis, rRNA synthesis generates a pre-rRNA transcript that includes mature rRNA (16S and 23S) and short-lived spacer sequences. Whereas mature rRNA is stable, spacer regions are rapidly degraded; therefore, the abundance of pre-rRNA relative to mature rRNA (also defined as rRNA synthesis ratio [RS ratio]) serves as a measure of ongoing rRNA synthesis. The RS ratio has been validated as a molecular biomarker that strongly correlates with growth rate of M. tuberculosis *in vitro* and *in vivo* in the absence of drug treatment (40).

**FIG 4:**
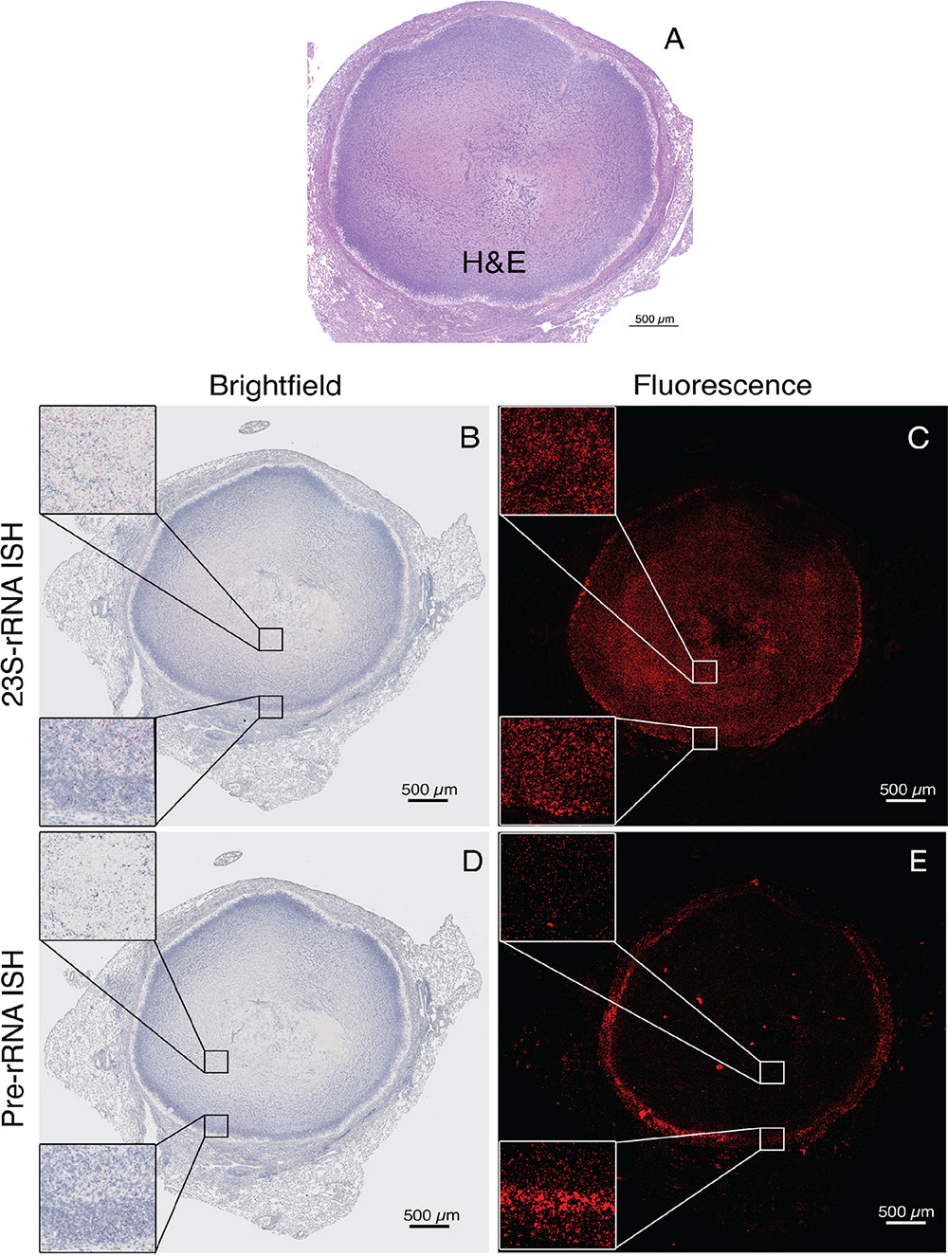
Visualization of replication status of individual bacteria in caseous necrotic type I lesions of infected C3HeB/FeJ mice, using ISH with pre-rRNA and 23S-rRNA probes. (A) Hematoxylin and eosin staining of a caseous necrotic type 1 pulmonary lesion from a Mycobacterium tuberculosis-infected C3HeB/FeJ mouse. (B to E) Two serial tissue sections stained by *in situ* hybridization for 23S-rRNA (B and C), visualizing all bacteria in both the cellular-caseum interface, and acellular region of the necrotic core (caseum) for precursor rRNA (D and E) visualizing actively replicating bacteria almost exclusively in the cellular region located on the caseum-rim interface. Signals were developed with a fluorescing chromophore allowing visualization by bright-field and fluorescence microscopy.

Using RNA *in situ* hybridization (ISH), both the pre-rRNA and 23S rRNA signals were detected in bacilli located in caseous necrotic type I lesions of untreated animals (Fig. 4). Visual comparison of pre-rRNA to the 23S rRNA signal intensities revealed population heterogeneity within both the cellular-caseum interface and in the acellular caseum core of type I lesions. An H&E-stained image of an adjacent section of the same lesion is shown for reference (Fig. 4A). The 23S-rRNA signal, as a marker for overall mycobacterial burden, showed a positive signal at similar densities in both the cellular region and the acellular region of the caseum core (Fig. 4B and C). The pre-rRNA signal, as a molecular biomarker for bacterial replication, was far more pronounced at the cellular-caseum interface where bacteria are mostly intracellular, harbored in foamy macrophages and neutrophils. The bacteria in the acellular core of type I lesions (caseum), where bacteria are primarily extracellular, only showed very weak pre-rRNA signals, indicating dramatically reduced new rRNA synthesis (Fig. 4D and E). In summary, rRNA synthesis was lower in caseum, indicating slowed M. tuberculosis rRNA synthesis, which, in the absence of any drug treatment, is consistent with a significantly reduced bacterial replication rate (40) in the hypoxic caseum core relative to the better-aerated cellular-caseum interface.

### Comparative pharmacokinetics of DprE1 inhibitors in C3HeB/FeJ mice.

**FIG 5:**
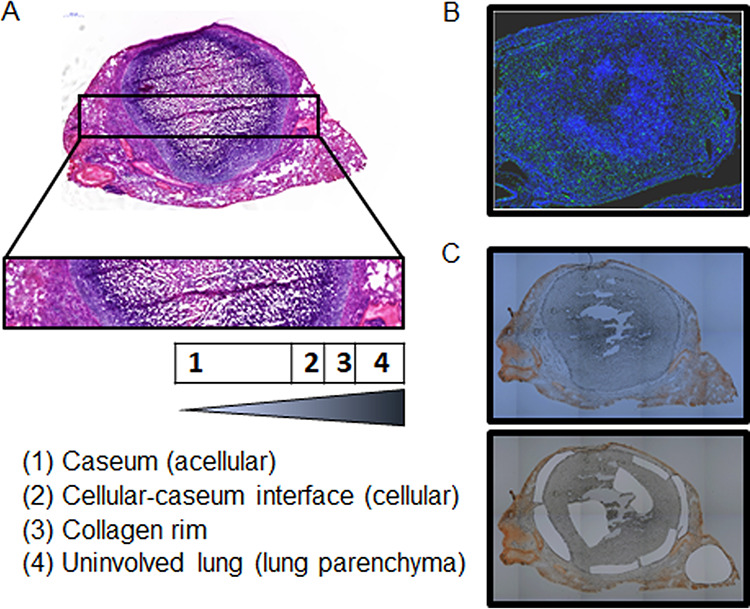
Typical compartments of a type I necrotic lesion in Mycobacterium tuberculosis-infected C3HeB/FeJ mouse lungs. (A) Visualization of various lesion areas on H&E-stained sections of a type I lesion. (B) SYBR gold staining of a type I lesion with high bacterial numbers (green fluorescent) located intracellularly in a foamy macrophage layer at the cellular-caseum interface as well as extracellular in caseum (acellular). (C) Serial section of a type I lesion before and after excision of lesion areas by laser capture microdissection (the adjacent section was stained with hematoxylin and eosin).

To study the relationship between exposure and activity (PK-PD) in lesions and how this correlates with efficacy, dedicated groups of C3HeB/FeJ mice were infected by low dose-aerosol with M. tuberculosis Erdman. Ten weeks postinfection, mice received the study compounds for two and a half weeks to reach steady state in tissues. Plasma samples and tissues were collected from mice for every treatment group at T1 (*T*_max_, or time of peak plasma concentration) and T2 (*T*_min_, or end of the dosing interval) based on published or reported PK studies in mice (9, 15, 18). T1 and T2 were 0.5 h and 12 h for TBA-7371, 15 min and 24 h for PBTZ169, and 1 h and 24 h for OPC-167832. Tissue drug levels were quantified by (i) standard high-pressure liquid chromatography coupled to tandem mass spectrometry (LC-MS/MS) in lung and lesion homogenates and (ii) gravity-assisted laser capture microdissection (LCM) in thin sections of specific lesion areas followed by LC-MS/MS. Four different areas were sampled by LCM within mature caseous necrotic pulmonary lesions: (i) caseum, consisting of cellular debris with high numbers of extracellular bacteria, showing limited replication by RS ratio (acellular); (ii) cellular-caseum interface, consisting of foamy macrophages and neutrophils with high numbers of intracellular, replicating bacteria (cellular); (iii) the collagen rim; and (iv) uninvolved lung (Fig. 5). Similar results were obtained by both quantitative measures. The LCM approach achieves accurate spatial drug quantitation in caseum and eliminates contamination by the foamy macrophage layer inside the collagen rim (41). A disadvantage of LCM, however, is the detection limit, which is generally 5- to 10-fold higher than that of the standard tissue PK method due to the higher dilution factor (41). Therefore, both methods were complementary and beneficial to this study.

**FIG 6:**
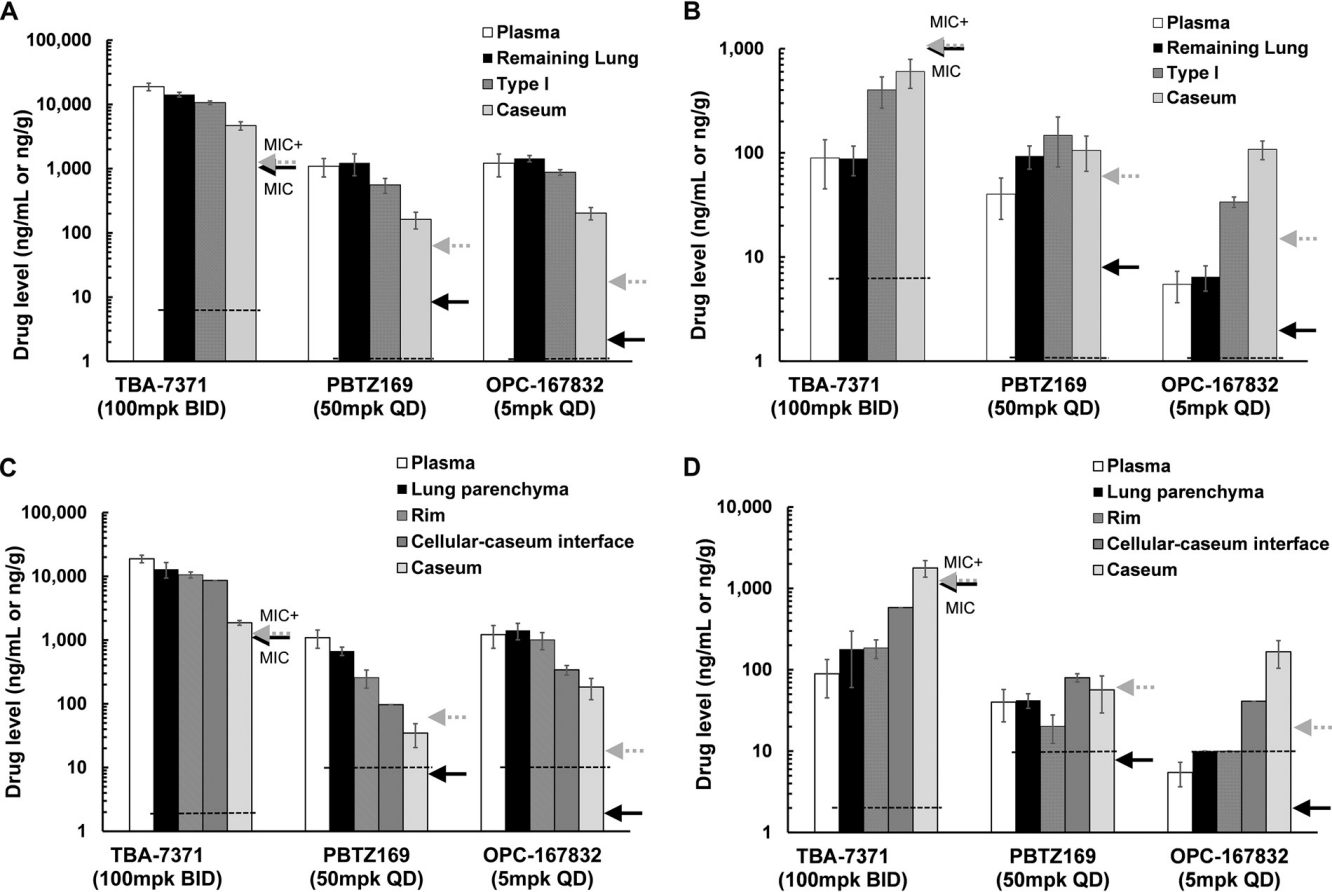
Determination of plasma and pulmonary lesion drug levels in Mycobacterium tuberculosis infected C3HeB/FeJ mice after 2.5 weeks of treatment with TBA-7371 (100 mg/kg, BID), PBTZ169 (50 mg/kg, QD), and OPC-167832 (5 mg/kg, QD). Pharmacokinetic results presented are for both standard lesion LC-MS/MS (A and B) and LCM-LC-MS/MS (C and D) approaches for quantification of DprE1 inhibitors in plasma and tissue samples, at plasma peak (A and C) or at trough time points (B and D) for TBA-7371 (at 0.5 and 12 h), PBTZ169 (at 0.25 h and 24 h), and OPC-167832 (at 1 h and 24 h), respectively. Drug levels determined by both approaches gave similar results, with high sensitivity of LCM-LC-MS/MS enabling accurate quantification within the caseum core with a lower detection limit. Arrows indicate MIC (in black) and MIC in the presence of 4% human serum albumin (MIC^+^, in gray) for the individual compounds. Detection limits of the analytical analysis method per compound are indicated with dotted horizontal lines.

At T1, a marked concentration gradient across the caseous lesions was observed for all three compounds, decreasing from uninvolved lung to the core of the necrotic lesion [from high to low, remaining lung > type I > caseum by standard tissue PK, or from high to low, uninvolved lung parenchyma > collagen rim > cellular-caseum interface [cellular] > caseum [acellular and nonvascularized] by LCM) (Fig. 6A and C). For all three compounds, drug levels were high and similar in plasma and whole lung and well above the MIC and the MIC in the presence of 4% human serum albumin (MIC+huSA) throughout the dosing interval. TBA-7371 reached 11 to 19 μg/ml or μg/g in plasma and lungs, respectively, 5- to 10-fold above the MIC+huSA, PBTZ169 achieved concentrations around 1 μg/ml or μg/g in plasma and lungs, respectively, 11- to 20-fold above the MIC+huSA, and OPC-167832 plasma and lung concentrations were 1.2 to 1.4 μg/ml or μg/g, 75- to 85-fold above the MIC+huSA. The superior PK of OPC-167832 was even more pronounced in the cellular-caseum interface (cellular) and caseum core (acellular), which both are primary pulmonary locations of bacteria in C3HeB/FeJ mice (Fig. 4). Drug levels in caseum were significantly lower than those in plasma or whole lung for all three inhibitors. In caseum, TBA-7371 concentrations were 4-fold higher than its MIC+huSA, PBTZ169 either failed to achieve or just covered the MIC+huSA by a narrow margin, and OPC-167832 concentrations were 20-fold in excess of the MIC+huSA. In caseum (acellular), only OPC-167832 achieved appreciable coverage of the MIC+huSA at T1, at 0.19 μg/g or 12-fold above the MIC+huSA (Fig. 6A and C).

At T2 (12 h for TBA-7371 and 24 h for the other compounds), a reversed concentration gradient was observed for TBA-7371 and OPC-167832 (Fig. 6B and D): from low to high, remaining lung < type I < caseum by standard tissue PK, or from low to high, uninvolved lung parenchyma < collagen rim < cellular-caseum interface (cellular) < caseum (acellular) by LCM, with the latter showing the best retention of both TBA-7371 and OPC-167832 at the end of the dosing interval. For PBTZ169, no gradient in drug levels was observed, resulting in similar drug levels across the various regions of the necrotic lesion and in plasma. At trough, TBA-7371 concentrations dropped below the MIC and MIC+huSA in plasma, lungs, and type I lesions and either failed to achieve or just covered the MIC in caseum by a narrow margin (Fig. 6B and D). PBTZ169 remained around the MIC+huSA in all compartments, or 6- to 13-fold over the MIC without supplemental huSA. OPC-167832 concentrations were 0.034 to 0.108 μg/g in type I lesions and in caseum, which is about 2- to 7-fold over the MIC+huSA and 17- to 54-fold over the MIC without supplemental huSA (Fig. 6B and D).

## DISCUSSION

Various drug discovery and development initiatives are currently ongoing to identify and develop new potent TB drugs with novel MOA for inclusion in new drug regimens to help shorten the length of TB treatment in patients (7, 8). One of the drug classes with a new MOA are DprE1 inhibitors targeting an essential process in cell wall synthesis (reviewed in reference 12). Most preclinical evaluation of DprE1 inhibitor efficacy as single agents and in regimens were performed in BALB/c or ICR mouse models (9, 15, 18), which do not develop hypoxic caseous necrotic lesions and where the majority of the bacteria reside in macrophages (27). The present study was designed to (i) assess side-by-side the efficacy of three novel clinical development candidates that target DprE1 in a single study and (ii) confirm the efficacy observed in BALB/c mice and study the site-of-disease PK/PD of these DprE1 inhibitors in a C3HeB/FeJ mouse model with heterogeneous bacterial populations caused by diverse pulmonary lesion environments. In the C3HeB/FeJ mouse model, the majority of bacteria are located in caseous necrotic type I pulmonary lesions, with a high burden of intracellular bacteria in the foamy macrophage layer and in neutrophils inside the collagen rim (cellular-caseum interface) and an even higher proportion of extracellular bacteria in the caseum core (23). To be efficacious in C3HeB/FeJ mice, anti-TB agents have to distribute throughout the caseous necrotic lesions and reach adequate exposure at these primary bacterial locations.

All three DprE1 inhibitors showed significant efficacy after 8 weeks of treatment 5 of 7 days per week in C3HeB/FeJ mice, with an average 1.5-log_10_ lung CFU reduction compared to untreated controls for TBA-7371 at 100 and 200 mg/kg BID and for PBTZ169 at 50 and 100 mg/kg QD and an average 3-log_10_ lung CFU reduction for OPC-167832 at 5 and 20 mg/kg QD. The confirmed efficacy in this more complex mouse model provides an additional preclinical data point for the further clinical development of this series. The LME in lungs for the three inhibitors in C3HeB/FeJ mice was the same as the MED_99_ from earlier mouse models without necrotic lesions. The LME in C3HeB/FeJ mice for TBA-7371 was determined as 100 mg/kg, BID, the LME for PBTZ169 was 50 mg/kg, QD, and the LME for OPC-167832 was 5 mg/kg, QD. Generally, the MED_99_ and LME values of lead compounds are integrated into comprehensive preclinical PK/PD data sets and used for human dose projections for phase I and II clinical studies. In spleens, which only present with cellular lesions without caseous necrosis in C3HeB/FeJ mice, both TBA-7371 and OPC-167832 showed similar efficacy at the medium and high dose levels. Although different mouse models were used for the preclinical development of each drug individually and, therefore, caution should be exercised when comparing the data, similar efficacy for TBA-7371 at 100 mg/kg and OPC-167832 at 5 mg/kg was observed in nonnecrotic preclinical murine models (16, 18). In C3HeB/FeJ mouse lungs, however, OPC-167832 showed a clear superiority. This underscores the potential benefit and importance of testing late-stage TB drug candidates in a mouse model with advanced lung pathology reflective of human disease.

OPC-167832 showed superior efficacy compared to the two other DprE1 inhibitors, with the highest kill rate in the first month of treatment, even at the low doses tested, The superior efficacy of OPC-167832 in C3HeB/FeJ mice can be attributed to a combination of potent *in vitro* antimicrobial activity, good *in vivo* drug exposure in plasma, lung, and lesions, and substantial retention of the compound in caseum at trough. The compound shows potent *in vitro* activity, with a MIC of 0.002 μg/ml, with about an 8-fold shift in MIC in the presence of physiologic levels of human serum albumin due to high protein binding. In both plasma and lung tissue, OPC-167832 displays good drug distribution and tissue penetration at the maximum concentration in serum (*C*_max_), with drug levels at steady state around 1 μg per ml (plasma) or g (tissue) after administration at 5 mg/kg. Drug levels at plasma *C*_max_ in the caseous core of necrotic lesions were significantly lower than those measured in plasma or whole lung (with 0.035 μg/g in the cellular-caseum interface) but were still approximately 21-fold in excess of the MIC+huSA. At trough, only OPC-167832 showed adequate drug exposure above the MIC+huSA in the cellular-caseum interface and in caseum of type I lesions, which is where the majority of bacteria are located in C3HeB/FeJ lungs. The retention of substantial drug levels over 24 h at the locations where the bacteria reside is arguably the most important beneficial attribute of the OPC-167832 compound.

While PBTZ169 was efficacious only in the second month, it showed one of the highest kill rates recorded in this study at both 50 and 100 mg/kg (*k* = 0.041 to 0.047; see Table S1A in the supplemental material) and was as efficacious at 50 mg/kg QD as TBA-7371 at 100 mg/kg BID. The basis for the difference in observed kill rates between the various DprE1 inhibitors is not known. This result shows that both covalent (PBTZ169) and noncovalent (TBA-7371) inhibitors of DprE1 can be efficacious *in vivo.* The high plasma protein binding of PBTZ169 (99.65%) may have contributed to delays in reaching steady state in lesions, translating into delayed efficacy. After 2.5 weeks of PBTZ169 dosing at 50 mg/kg, *C*_max_ of approximately 1 μg/ml or μg/g, 11- to 20-fold above the MIC+huSA, was reached in plasma and lungs. In contrast, *C*_max_ in the cellular-caseum interface and caseum were 10-fold lower than plasma or whole lung *C*_max_, barely above the MIC+huSA, which could explain its moderate efficacy. Due to its high protein binding, PBTZ169 showed a low peak-to-trough range in lesions, achieving almost constant drug levels around the MIC+huSA and consistent drug levels across the various lesion compartments and in plasma at trough. A potential caveat in exclusively measuring the parent PBTZ169 is the presence of potentially active metabolites. Recently, Kloss et al. reported on the occurrence of a novel active metabolite, H_2_-PBTZ169 (42). H_2_-PBTZ169 is biologically active and unstable and easily reverts to PBTZ169 upon air oxidation, which could affect the quantitation of the parent PBTZ169 and may explain occasional discrepancies between concentrations measured by laser capture microdissection (LCM) of thin lesion sections and in lesion homogenates. Overall, PBTZ169 concentrations were lower in LCM samples, which requires handling at room temperature during the dissection procedure. Although new ultrahigh-performance LC-MS/MS methods have been recently developed to simultaneously detect PBTZ169 and its 5 metabolites (43), the accurate quantification of this molecule in biological fluids is more challenging than originally thought.

TBA-7371 showed significant efficacy compared to the untreated controls and a moderate, consistent kill rate throughout the treatment duration. At 100 mg/kg BID administration for 2 months, TBA-7371 was as efficacious as PBTZ169 at 50 mg/kg QD, despite having the highest MIC (1 μg/ml against M. tuberculosis Erdman) of all three DprE1 inhibitors with no appreciable shift in MIC in the presence of 4% human serum albumin. TBA-7371 showed a *C*_max_ values around 11 to 19 μg/ml or μg/g in lungs and plasma, which were 5- to 10-fold above the MIC. As observed with all DprE1 inhibitors, TBA-7371 *C*_max_ values in the cellular-caseum interface and caseum were significantly lower than in plasma or whole lung, around 8.6 μg/g in the cellular-caseum interface, 4-fold above the MIC+huSA. At trough, a reverse concentration gradient was observed in necrotic lesions, with the lowest drug levels in uninvolved lung parenchyma. The short half-life and low protein binding of TBA-7371 likely contributed to poor retention in caseum at the end of the dosing interval (12 h), which was barely over the MIC. This could explain the moderate efficacy of TBA-7371 in the C3HeB/FeJ mouse model.

Although the frequency of spontaneous resistance development is quite low and similar for the three DprE1 inhibitors tested here (i.e., <10^−7^), we did not evaluate potential emergence of drug resistance during treatment *in vivo*, which is a potential limitation of this efficacy study.

Histopathology analysis using two complementary approaches (i.e., evaluation by a board-certified pathologist and by pathologist-assistive software, called LIRA) confirmed that all three DprE1 inhibitors were able to halt the progression of pulmonary pathology from treatment start by preventing the development of destructive type II neutrophilic lesions. A higher incidence of type III cellular lesions in PBTZ169-treated mice was observed than in the untreated control group, which could be due to the delayed treatment response of PBTZ169 in C3HeB/FeJ mice, where efficacy was only seen after 2 months of treatment. Increased inflammation has been described early in treatment when high bacterial numbers are killed, causing an immunogenic response (44, 45). A more comprehensive histopathology study would be required to confirm the significance of this result.

Cell wall synthesis inhibitors are primarily active against replicating bacteria, an intrinsic consequence of their MOA (46). Accordingly, all three DprE1 inhibitors showed good kill kinetics (Fig. 1B) *in vitro* against replicating bacteria but were inactive at physiologic concentrations measured in mouse lungs against nonreplicating M. tuberculosis either cultivated *in vitro* under hypoxic conditions (Fig. 1D) or in rabbit caseum (Fig. 1C). To assess the replication status of M. tuberculosis in C3HeB/FeJ mouse lungs, we used quantitative multiplexed RNA-*in situ* hybridization (ISH) to measure pre-rRNA and 23S rRNA signals in bacteria in sections of caseous necrotic type I lesions of untreated animals (40). Results demonstrated that bacterial populations in the C3HeB/FeJ mouse model producing new rRNA, a biomarker associated with active bacterial replication (40), are primarily located in the foamy macrophage and neutrophil layers directly inside the collagen rim in necrotic lesions (i.e., cellular-caseum interface; Fig. 4D and E). Both cell types appear highly permissive to M. tuberculosis infection and seem no longer functional in controlling bacterial growth. This was in contrast to extracellular bacteria in caseum core, shown to be nonreplicating by ISH, resulting in a substantially reduced signal for pre-rRNA versus 23S rRNA markers. Direct evidence of nonreplicating bacteria in caseum was lacking until a recent publication characterized bacteria extracted from TB infected rabbit caseum, subsequently cultured for growth and measured for chromosomal equivalents (47). Thus, drug exposure and effect (PK/PD) in the foamy macrophage and neutrophil layers (cellular-caseum interface) of the type I lesions are likely the main contributors to DprE1 inhibitor efficacy in C3HeB/FeJ mice. This observation implies that drug distribution into the cellular layers inside the necrotic lesion, where replicating M. tuberculosis thrives, is a requisite and a key factor in eliciting an effective drug treatment response in this mouse model. In contrast, drug levels in the acellular caseum compartment are likely not contributing to DprE1 inhibitor efficacy. This hypothesis could be tested by assessing bacterial viability within surgically dissected lesion compartments or using the RS ratio *in situ* hybridization approach in C3HeB/FeJ mice following drug treatment.

In conclusion, we have demonstrated efficacy of three DprE1 inhibitors in the C3HeB/FeJ mouse model after 2 months of treatment. Superior efficacy was observed for OPC-167832, even at low doses, which can be attributed to its low MIC, good drug distribution, and sustained retention above the MIC throughout the dosing interval in necrotic lesions where most bacteria reside in M. tuberculosis-infected C3HeB/FeJ mice. As more clinical trial data become available, these attractive drug candidates constitute a necessary addition to novel TB drug regimens. These studies also highlight the utility of newer LCM- and ISH-based approaches to provide insight into lesional PK/PD parameters and pathology classification, especially for anti-TB drugs in late-stage development.

## MATERIALS AND METHODS

### *In vitro* activity testing.

M. tuberculosis H37Rv strain (Trudeau Institute, Saranac Lake, NY) and M. tuberculosis strain Erdman (TMCC 107) were both grown in Proskauer-Beck liquid medium containing 0.05% Tween 80 (Sigma-Aldrich, St. Louis, MO) to mid-log phase, aliquoted, and frozen at −80°C until use in the *in vitro* assays.

The MIC was determined for TBA-7371, OPC-167832, and PBTZ169 against M. tuberculosis Erdman and M. tuberculosis H37Rv in 7H9 medium supplemented with 0.2% glycerol and 10% ADC without Tween 80. MICs were determined by a broth microdilution assay using 2-fold serial drug dilutions with an alamarBlue endpoint (MABA) (48, 49). The lowest consecutive antimicrobial concentration that did not produce visible color change with alamarBlue and/or showed a ≥80% reduction in optical density at 600 nm (OD_600_) relative to drug-free control wells was regarded as the MIC. In parallel, the MICs were also determined in the presence of 4% (wt/vol) human serum albumin (huSA) (number A1653; Sigma) to assess potential protein binding (serum-shift assay). Generally, a shift in MIC of two wells (4-fold shift in MIC) is considered a significant shift indicating protein binding. For pretomanid (positive control), a 4- to 8-fold shift in MIC is to be expected.

The kill kinetics of the three DprE1 inhibitors against M. tuberculosis Erdman were determined under replicating conditions at 37°C in 7H9 medium supplemented with 0.2% glycerol, 10% ADC, and 0.05% Tween 80 (Sigma-Aldrich, St. Louis, MO). Individual DprE1 inhibitors were added to actively growing cultures (2.2 × 10^5^ CFU/ml) at 4× their respective MIC. An equal volume of dimethyl sulfoxide (DMSO) (i.e., 1%) was included as a vehicle control for comparison. The numbers of surviving CFU/ml were determined over 14 days by serial dilution and culture on Middlebrook 7H11 agar plates supplemented with 0.2% glycerol, 10% oleic acid-albumin-dextrose-catalase (OADC) supplement (GIBCO BRL, Gaithersburg, MD), 0.01 mg/ml cycloheximide, and 0.05 mg/ml carbenicillin (defined here as 7H11 agar plates), which was further supplemented with 0.4% activated charcoal to prevent drug carryover, as described previously (50).

To test the *in vitro* activity of the three DprE1 inhibitors against M. tuberculosis grown under oxygen-depleting conditions, the rapid anaerobic dormancy (RAD) assay was performed (38). Briefly, aliquots of frozen bacteria were used to start aerobic cultures in Dubos medium (Becton-Dickinson, Sparks, MD) prepared according to the manufacturer’s directions and supplemented with 0.05% Tween 80. Aerobic cultures were grown in 10-ml volumes in 150- by  25-mm screw cap tubes at 37°C with rapid stirring for 7 to 10 days until reaching mid-log phase (OD_600_ of 0.4 to 0.6). Cultures were then diluted 1:100 in Dubos Tween-albumin medium to an OD_600_ of 0.005 in 15-mm by 125-mm screw cap tubes containing stir bars (12 mm by 4.5 mm) at a culture-to-headspace ratio of 0.65 (adjusted for the Colorado altitude, compared to a ratio of 0.5 for sea level). Cultures were stirred rapidly (200 rpm) using a rotary magnetic tumble stirrer (V & P Scientific, San Diego, CA). To three tubes, 30 μl of methylene blue stock solution at 500 μg/ml was added (final concentration of 1.5 μg/ml) as a control for visual confirmation of anaerobic conditions, as methylene blue decolorizes with oxygen depletion (51). TBA-7371 was tested at 1×, 10×, and 100× MIC (1, 10, and 100 μg/ml); PBTZ169 was tested at 1×, 10×, 100×, and 1,000× MIC (0.008, 0.08, 0.8, and 8 μg/ml); and OPC-167832 was tested at 1×, 10×, 100×, and 1,000× MIC (0.002, 0.02, 0.2, and 2 μg/ml). Control drugs included isoniazid (INH) at 10 μg/ml, rifampin (RIF) at 10 μg/ml, and ethambutol (EMB) at 100 μg/ml. Drugs were dissolved in 100% DMSO at 100× final concentration. Drugs were added to 8-day-old anaerobic cultures by injection through the rubber septa in a 100-μl volume. Drug exposure lasted either 5 days or 8 days, after which the bacterial suspensions were diluted and plated onto 7H11 agar plates. All compounds were tested in duplicate tubes for both time points. After 5 days of drug treatment, cultures were resuspended and 100-μl portions were taken from the culture tubes using a tuberculin syringe (number 309301, 3/10 cc, 29 gauge, 1/2 inch; BD) to avoid introducing oxygen to the cultures; 50 μl of the sample was used for serial dilutions and plating onto 7H11 agar plates. After 8 days of drug treatment, the tubes were uncapped and the samples resuspended using a serological pipette, and serial dilutions of the cultures were plated onto 7H11 agar plates. Plates were incubated under normal atmospheric conditions, and bacterial colonies were enumerated after ≥21 days of incubation at 37°C.

The caseum MBC assay was performed to assess the activity of OPC-167832 against nonreplicating bacteria in rabbit caseum, as described previously (52). Briefly, each drug stock solution (50 mM) was serially diluted in the corresponding vehicle, and 1 μl of each dilution was dispensed in each well of a 96-well assay plate. OPC-167832 was evaluated at the final concentration range of 0.03 to 1,000 μM. Frozen caseum extracted from rabbit lungs was thawed and homogenized in 2 volumes of sterile water using a Fastprep-24 instrument (MP Biomedicals) and 1.4-mm-diameter ceramic (zirconium oxide) beads. Fifty microliters of caseum homogenate was dispensed in each well. The 96-well plates were incubated at 37°C for 7 days. After incubation, caseum homogenate from each well was serially diluted and plated on 7H11 agar plates in triplicate. Agar plates were incubated for up to 4 weeks before CFU counts were performed. The caseum MBC_90_ was defined as the concentration of drug required to achieve killing of 90% of M. tuberculosis bacteria contained in the caseum sample. The statistical significance of the differences in CFU counts between day 0 and day 7 (DMSO-only controls) was analyzed using paired *t* tests.

### Animals.

Female specific-pathogen-free C3HeB/FeJ mice, aged 8 to 10 weeks, were purchased from Jackson Laboratories (Bar Harbor, ME). Mice were housed in an animal biosafety level III (ABSL-3) facility, employing autoclaved bedding, water, and mouse chow *ad libitum*. Specific-pathogen-free status was verified by testing sentinel mice housed within the colony. All animal studies were performed at Colorado State University in a certified ABSL-3 facility in strict accordance to the regulations and recommendations of the *Guide for the Care and Use of Laboratory Animals* of the National Institutes of Health and the Centers for Disease Control and Prevention (53). All procedures and performed protocols for infecting mice with M. tuberculosis and subsequent drug treatments in the described mouse infection studies were approved by the Colorado State University Institutional Animal Care and Use Committee (IACUC) (reference numbers of approved protocols: 09-1367A, 12-3723A, 15-5942A, and 18-8006A). All experiments were approved by the IACUC prior to initiation of mouse studies and conducted by following the relevant guidelines and regulations. Mice were euthanized by CO_2_ inhalation, a method approved by the CSU IACUC.

### Aerosol infection.

Mice were infected by aerosol with the M. tuberculosis Erdman strain (TMCC 107), and the infective bacterial inocula were prepared as described earlier (54). Briefly, bacteria were initially grown as a pellicle to generate low-passage-number seed lots of virulent bacteria. Working stocks were generated by growing a proportion of the seed lot to mid-log phase in Proskauer-Beck medium containing 0.05% Tween 80 (Sigma Chemical Co., St. Louis, MO) using three passages, enumerated by colony enumeration on 7H11 agar plates, divided into 1.5-ml aliquots, and stored at −70°C until use. C3HeB/FeJ mice were exposed to a low-dose aerosol infection using a Glas-Col inhalation exposure system, as previously described (55), resulting in an average of 70 bacteria in the lungs per mouse on the day of exposure. Five mice per aerosol run were sacrificed the following day to determine the number of CFU implanted in the lungs for a total of three aerosol runs. Infected mice were observed and weighed at least once a week. Starting at day 21 until the start of therapy, mice were observed and weighed two to three times per week due to the increased incidence of morbidity and mortality associated with clinical TB disease. Any mice exhibiting clinical symptoms of illness were humanely euthanized. For every treatment group, eight mice per group were used to achieve sufficient statistical power in this mouse model.

### Drug efficacy experiments and bacterial enumeration.

For drug efficacy experiments, C3HeB/FeJ mice from different aerosol runs were randomized (*n* = 8 per group per time point) and dosed starting 8 weeks after aerosol with TBA-7371 (at 50, 100, and 200 mg/kg, BID), PBTZ169 (at 25, 50, and 100 mg/kg, QD), or OPC-167832 (at 1.25, 5, and 20 mg/kg, QD). Treatment occurred 5 out of 7 days per week (Monday through Friday) for a total of 8 weeks. TBA-7371 (free base) was formulated weekly in 0.5% carboxy-methylcellulose (CMC) (Sigma-Aldrich, St. Louis, MO, USA) plus 0.1% Tween 80 and stored at 4°C until use. PBTZ169 (HCl salt) was formulated in 0.5% CMC by first grinding the powder in a mortar with a pestle and gently adding drops of 0.5% CMC (pH   7.0). The PBTZ169 suspension was stored at 4°C and, prior to use, acclimated to ambient room temperature (RT) and vortexed for 1 min to achieve a homogenous suspension. OPC-167832 was formulated weekly in a 5% (wt/vol) gum Arabic solution (Sigma-Aldrich, St. Louis, MO, USA) by grinding the compound in an agate mortar with a pestle and then gently adding dropwise the 5% (wt/vol) gum Arabic solution from a polystyrene bottle using a plastic dropper. The gum Arabic solution was prepared by mixing gum Arabic powder (5% [wt/vol]) with deionized water, heated at 90 to 95°C in the water bath, and then stirring at room temperature for 1 to 2 h to ensure complete dissolution. The OPC-167832 suspension was sonicated for 5 to 10 min in a water bath and stored at 4°C until use.

At the time of sacrifice, whole lungs from eight mice per treatment group were aseptically removed and disrupted with a tissue homogenizer (Precellys; Bertin Instruments, Rockville, MD). An additional four mice of the high-dose group for every treatment were sacrificed after 8 weeks and lungs fixed for histopathology purposes (described below). To assess efficacy, the number of viable organisms was determined by plating serial dilutions of whole lungs homogenized in 4 ml of phosphate-buffered saline (PBS) on 7H11 agar plates. Due to the long half-life and high protein binding capacity of both PBTZ169 and OPC-167832, lungs and spleens from all drug-treated animals as well as untreated controls were homogenized in PBS plus 10% bovine serum albumin and plated on 7H11 agar further supplemented with 0.4% activated charcoal to prevent drug carryover as described previously (50). Colonies were enumerated after at least 28 days of incubation at 37°C, and plates were incubated for 8 weeks to ensure all viable colonies were detected.

### Statistical analysis.

The viable CFU values were transformed to logarithms as log_10_ CFU per organ, which were then first evaluated by a one-way analysis of variance (ANOVA) of the treatment groups versus untreated controls either at the start or end of treatment. Results were also evaluated by a two-way ANOVA, with adjustments for multiple comparison using one-way Tukey’s test (pairwise comparison between all treatment groups) or Dunnett’s test (for comparison of each treatment to the untreated or pretreatment controls) using Prism 8 (GraphPad Software, San Diego, CA). Differences were considered significant at the 95% level of confidence. Finally, a three-way ANOVA analysis was performed on log-transformed CFU data from lung and spleen tissues for differences based on drug treatment (OPC-167832, TBA-7371, and PBTZ169) and dosage (low, medium, or high) at two time points (4 and 8 weeks). Log_10_ CFU numbers were modeled with linear regression, including interactions between drugs and doses including both time points, separately for each tissue type. Pairwise comparisons of the group means were evaluated using estimated marginal means calculated with the R package emmeans version 1.3.4.1. *P* values were adjusted with a Tukey method for comparing a family of 9 estimates. Linear regressions were conducted in R version 3.6.0 (2019-04-26).

### Histological analysis.

A separate group of four mice at the high-dose level for every treatment was sacrificed after 8 weeks, and whole lungs were fixed for histopathology purposes. Lung perfusions were performed by clamping the caudal vena cava with straight-tipped hemostats to prevent 4% paraformaldehyde (PFA) from escaping into the abdominal cavity and by clamping the cranial vena cava with to clamp to retain PFA in the lungs. A scalpel was used to make a small incision into the left ventricle of the heart for blood to drain, and a syringe with a 26-guage, 1/2-inch needle was used to inject slowly 10 ml of 4% paraformaldehyde into the right ventricle for lung fixation. Lungs were then placed into a designated histology cassette into a jar of 4% PFA to fix for 48 h. After 48 h, lungs were placed into fresh 1× PBS. Whole lungs were sectioned and stained with H&E. Digital image scans from microscopic slides containing the C3HeB/FeJ lungs were generated at Premier Laboratories (Longmont, CO) on an Aperio Scanscope XT digital slide scanner (Nikon, Melville, NY), at ×20 magnification, with an image resolution of 0.5 μm/pixel.

Histopathology analysis was performed using two different, complementary approaches. The first classical pathology analysis was performed blindly by a board-certified pathologist, specialized in M. tuberculosis pathology examination in mouse and Guinea pig models (Brendan Podell, DMIP, CSU), using semiquantitative scoring of lung involvement per lesion type as well as a qualitative analysis describing the specific pathology features for every slide. The manual histopathology analysis by a pathologist used a system of scoring of lung involvement (56). Mouse sections were provided a semiquantitative pathology score based on three major categories, including extent and frequency of granulomas, the cellular composition of granulomas, and the degree of destructive inflammatory pathology. For extent and frequency of granuloma lesions, lungs of mice were assigned a score of 1 to 4 based on an overall burden of <10%, 10 to 25%, 25 to 50%, or >50%, respectively. In evaluating cellular composition of lesions, lungs with lesions containing a higher frequency of lymphocytes were assigned a more favorable score of 1, while a mix of macrophages and lymphocytes with few neutrophils received a score of 2, and lungs with high proportions of macrophages and neutrophils received a score of 3. Destructive inflammatory pathology was scored on a scale of 1 to 3, based on the presence of well-delineated granulomas, significant extension of inflammatory cells into surrounding alveolar spaces or airway walls, or extensive inflammation along with individual cell or foci of necrosis and accumulation of proteinaceous fluid or fibrin. Collectively, a scale of severity was based on a minimum score of 1 and a maximum score of 10 for each animal. Data were expressed as mean score for each treatment group ± standard deviations (SD).

The second histopathology method used a newly developed pathologist-assistive software called LIRA (lesion image recognition and analysis [21]), which reduces user limitations in terms of time and reproducibility while providing a rapid quantitative scoring system for digital histopathology image analysis. The LIRA source code with accompanying documentation has been made available at https://Github.com/TB-imaging/LIRA. The LIRA software was developed as a modular neural network to identify the various pulmonary lesion types of C3HeB/FeJ mice. Briefly, after a digital image is uploaded, the macro-classifier (CNN1) identifies the type I lesion physiological structure with its characteristic caseous necrotic center and collagen rim. Human feedback is required after this initial step to ensure accurate classification. The macro-classifier CNN1 will then identify the individual image areas either positively or negatively for the type I pathology features. Based upon this macro-classifier CNN1 prediction, the digital image areas are partitioned into two separate data sets, classified as type I or not type I lesion. In case a type I lesion is detected after CNN1, CNN2 analyzes the image patches further and classifies each image patch as either type I rim, type I core, healthy tissue, miscellaneous, or empty. The remaining image patches (not identified by CNN1) are in parallel further classified with CNN3 as either type II, type III, healthy, miscellaneous, or empty. The output of the implementation of the three CNNs use raw image patch counts (RIPC) per classification category, which are sums of the tiled image patches for each classification. RPIC are classified and classification locations stored and subsequently visualized as colored overlays on the digital image (Fig. 3C). Every classification category is represented by a different color for easy visual qualitative analysis and result verification. The digital image of the lung lobe with colored overlay is then verified by the user at a second human intervention checkpoint. Ultimately, the final output of LIRA using the two human intervention checkpoints includes the enumeration of the various lesion types as well as the calculation of the area of lung involvement per classification category (presented as RIPC and percent lung involvement per category).

### *In situ* detection of 23S and pre-rRNA in C3HeB/FeJ lesions.

Mouse lungs were fixed with formaldehyde, paraffin embedded, and stained by multiplexed-ISH using optimized protocols, as previously described (39, 40). Briefly, mouse lungs were directly placed into 4% PFA prepared in 1× PBS upon excision, fixed for 48 h at 4°C, and paraffin embedded. Next, 2-μm sections were cut from formalin-fixed paraffin-embedded (FFPE) blocks and mounted onto Superfrost Plus microscope slides (Fisher Scientific). ISH for M. tuberculosis 23S and pre-rRNA was carried out by RNAscope (Advanced Cell Diagnostics), which was performed as described earlier (39, 40). Sequences for the M. tuberculosis-rRNA ISH probes were previously described (40). Whole-slide digital images were acquired at ×40 magnification using an Axio Scan Z1 slide scanning fluorescence microscope (Zeiss).

### Collection of samples for pharmacokinetic analysis.

For the PK experiments, C3HeB/FeJ mice (*n* = 8 per treatment group per time point) were dosed starting 10 weeks postaerosol with TBA-7371 (at 100 mg/kg, BID), PBTZ169 (at 50 mg/kg, QD), or OPC-167832 (at 5 mg/kg, QD). Treatment occurred 5 out of 7 days per week (Monday through Friday) for a total of 2.5 weeks to reach steady-state drug levels. Drugs were prepared as described for the efficacy studies above. Mice were euthanized, and plasma and tissues were collected at two time points, selected based on the plasma *C*_max_ and minimum concentration of drug in serum (*C*_min_) time points reported to us from past PK studies by the various research groups, further referred to here as T1 and T2. The T1 and T2 time points of plasma and tissue collections for the three compounds were the following: TBA-7371 at 0.5 h and 12 h, PBTZ169 at 15 min and 24 h, and OPC-167832 at 1 h and 24 h, respectively.

Whole blood was obtained via cardiac puncture and processed in plasma separator tubes (Becton, Dickinson and Co., Franklin Lakes, NJ), centrifuged at 10,000 relative centrifugal force for 2 min at 4°C, aliquoted into Eppendorf microcentrifuge tubes, and stored at −80°C until analysis. Mice with pronounced lung pathology were first selected to collect samples for spatial drug quantitation by gravity-assisted laser capture microdissection (LCM). Briefly, whole-lung samples consisting of the cranial, medial, and accessory lung lobes were collected on clear disposable base molds (Fisher Scientific, Hampton, NH, USA). Using forceps, tissues collected on trays were held in liquid nitrogen vapor over a Styrofoam cooler and frozen within 1 to 2 min. Tissue trays containing frozen lobes were wrapped in foil squares, placed individually into labeled Ziploc bags, and immediately transferred onto dry ice. The remaining mice were used for standard tissue PK and drug quantitation in lung and lesion homogenates. The left lobe and right caudal lobe were collected, and histologically uninvolved lung tissue identified by visual inspection was collected into tissue homogenization tubes (Cayman Chemical Company, Ann Arbor, MI, USA). Encapsulated caseous necrotic granulomas (type I) and caseum samples were similarly collected after careful dissection. Samples were stored at −80°C until analysis.

### Drug quantitation by LC-MS/MS.

Drug levels in tissue were determined using two complementary approaches: (i) by drug quantitation in plasma and tissue homogenates from dissected lungs, individually excised type I lesions, and/or caseum, by LC-MS/MS, and (ii) by spatial quantitation in thin tissue sections by LCM followed by LC-MS/MS analysis of microdissected areas (41). The benefit of the LCM approach is the ability to obtain absolute drug levels in defined areas of type I lung lesions (23) such as caseum (the core of the caseous necrotic lesion), foamy macrophage rim, necrotic neutrophil rim, and uninvolved lung without cross-contamination.

For standard tissue PK analysis, individual type I lesions and visibly uninvolved lung tissue pieces were weighed and homogenized in 10 volumes of PBS. Homogenization was achieved using a FastPrep-24 instrument (MP Biomedicals) and 1.4-mm zirconium oxide beads (Bertin Corp.). Neat 1-mg/ml DMSO stocks of all compounds were serially diluted in 50:50 acetonitrile (ACN)-H_2_O to create standard curves and quality control spiking solutions. Twenty microliters of neat spiking solutions was added to 180 μl of drug-free plasma or control tissue homogenate to create standards and QCs. Drug-free CD-1 mouse K_2_-EDTA plasma and lungs from BioIVT were used to build standard curves. Protein precipitation extraction (PPT) was performed by adding 10 volumes of solvent containing internal standard (IS) to 1 volume of plasma or homogenized tissue sample. Verapamil, PBTZ169-d11, and OPC-167832-d6 were used as the internal standards for TBA-7371, PBTZ169, and OPC-167832, respectively. ACN, methanol (MeOH), and 1:1 ACN-MeOH were used as PPT solvents for PBTZ169, OPC-167832, and TBA-7371, respectively. The PPT mixtures were vortexed for 5 min and centrifuged at 4,000 rpm for 5 min. The supernatant was then transferred for LC-MS/MS analysis.

LC-MS/MS analysis was performed on a Sciex Applied Biosystems Qtrap 6500+ triple-quadrupole mass spectrometer coupled to a Shimadzu Nexera X2 HPLC to quantify the clinical samples. Chromatography was performed with an Agilent Zorbax SB-C_8_ column (2.1 by 30 mm; particle size, 3.5 μm) using a reverse-phase gradient elution. Gradients used 0.1% formic acid in Milli-Q deionized water for the aqueous mobile phase and 0.1% formic acid in acetonitrile for the organic mobile phase. Multiple-reaction monitoring (MRM) of parent/daughter transitions in electrospray positive-ionization mode was used to quantify the analytes. The following MRM transitions were used for PBTZ169, PBTZ169-d11, OPC-167832, OPC-167832-d6, TBA-7371, and verapamil: 457.09/344.00, 468.16/344.00, 457.00/176.00, 463.10/182.10, 356.200/295.00, and 455.400/165.200, respectively. Sample analysis was accepted if the concentrations of the quality control samples were within 20% of the nominal concentration. Data processing was performed using Analyst software (version 1.6.2; Applied Biosystems Sciex).

For gravity-assisted LCM, lung samples containing type I lesions were first γ-irradiated in a Co-60 γ-irradiator to sterilize the tissues and eliminate all viable M. tuberculosis bacilli, using a validated procedure approved by the Institutional Biosafety Committee of Hackensack Meridian Health (41). Stability of the study compounds upon gamma irradiation was determined by comparing drug levels in lungs with and without irradiation from uninfected mice that had received a single dose of each compound. Serial sections of type 1 lesions were taken at 25 μm using a Leica CM1860 cryostat and mounted onto thin polymer membrane LCM slides (PET or PEN). Slides were placed on the Leica LMD 7000 microdissection microscope, and small user-defined areas of uninvolved lung, neutrophil and macrophage rich rims, and caseous centers were dissected using a UV laser. The captured areas of tissue were collected in individual tube caps sited below the specified regions of interest. These tissue areas were individually extracted and analyzed by LC-MS/MS for drug concentrations; 3,000,000 μm^2^ (3 mm^2^) of tissue surface area was sufficient for drug quantification by LC-MS/MS. LCM samples used identical extraction solvents and LC-MS/MS parameters as specified for plasma and homogenized tissues.

### Calculations for drug rate of killing in lungs and spleens of C3HeB/FeJ mice.

The drug kill rates for the observed lung and spleen CFU counts were calculated as the slopes of linear regression lines on the individual log_10_(CFU) values using

$${\text{log}}_{\text{1}0}\left[\text{CFU}\left(t\right)\right]\;=\;-k\cdot \;t\;+{\text{ log}}_{\text{1}0}\left[\text{CFU}\left(0\right)\right]$$

where *t* ≥ 0 is the time since start of treatment, *k* is the kill rate constant, and CFU(0) is the pretreatment value. The kill rates represent the daily rate of decrease in log_10_ CFU (with negative values representing an increase, or growth) and were calculated for the separate intervals, day 0 to day 28 (*k*_0−28_), day 28 to day 56 (*k*_28−56_), and day 0 to day 56 (*k*_0−56_), where the latter included all values for days 0, 28, and 56 simultaneously. The kill rates for DprE1 inhibitors in C3HeB/FeJ mice are shown in Table S1A and C in the supplemental material. Table S1B shows drug kill rates for type I and type III subsets identified for lung tissues.
