# Supplementary material for: Comparative Analysis of Pharmacodynamics in the C3HeB/FeJ Mouse Tuberculosis Model for DprE1 Inhibitors TBA-7371, PBTZ169, and OPC-167832
Source: Antimicrob Agents Chemother. 2021 Oct 18;65(11):e00583-21. doi: 10.1128/AAC.00583-21 (PMC8522729; doi:10.1128/AAC.00583-21)
Supplement: Supplemental file 1 — Supplemental Tables S1 and S2. Download AAC.00583-21-s0001.pdf, PDF file, 0.2 MB [file aac.00583-21-s0001.pdf]

SUPPLEMENTAL DATA

Suppl. Table 1: Kill rates for DprE1 inhibitors in C3HeB/FeJ mice after 4 and 8 weeks of treatment with TBA-7371 (at 50, 100, 200 mg/kg, BID), PBTZ169 (at 25, 50, 100 mg/kg, QD) and OPC-167832 (at 1.25, 5, 20 mg/kg, QD). Data represent the rate of killing and the standard error for the least squares fit to the data (SE) per day for the treatment periods [day x to day y], in log_{10}CFU per day. (**A**) Drug kill rates per day in lungs, (**B**) Drug kill rates per day in Type I (T1) and Type III (TIII) lesions in lungs, (**C**) Drug kill rates per day in spleens.

A

Drug (mg/kg)	k_[0-28]	k_[28-56]	k_[0-56]
PreRx & Untreated	-0.031 (0.014)	-0.015 (0.013)	-0.023 (0.006)
TBA-7371 (50)	-0.014 (0.012)	-0.001 (0.015)	-0.008 (0.007)
TBA-7371 (100)	0.000 (0.014)	0.021 (0.018)	0.011 (0.008)
TBA-7371 (200)	0.008 (0.013)	-0.001 (0.015)	0.004 (0.007)
PBTZ169 (25)	-0.025 (0.012)	0.005 (0.016)	-0.010 (0.007)
PBTZ169 (50)	-0.037 (0.012)	0.047 (0.013)	0.004 (0.008)
PBTZ169 (100)	-0.030 (0.011)	0.041 (0.014)	0.005 (0.008)
OPC-167832 (1.25)	0.016 (0.010)	-0.001 (0.011)	0.008 (0.005)
OPC-167832 (5)	0.032 (0.015)	0.024 (0.018)	0.028 (0.008)
OPC-167832 (20)	0.052 (0.015)	0.010 (0.020)	0.031 (0.009)

B

Drug (dose mg/kg)		k_[0-28]	k_[28-56]	k_[0-56]
PreRx & Untreated	TI	-0.019 (0.012)	-0.003 (0.007)	-0.010 (0.004)
	TIII	-0.007 (0.008)	-0.035 (0.014)	-0.020 (0.005)
TBA-7371 (50)	TI	0.001 (0.012)	-0.017 (0.010)	-0.009 (0.006)
	TIII	0.003 (0.006)	0.004 (0.007)	-0.001 (0.003)
TBA-7371 (100)	TI	0.010 (0.010)	0.004 (0.013)	0.007 (0.007)
	TIII	0.027 (0.006)	0.008 (0.013)	0.017 (0.004)
TBA-7371 (200)	TI	0.026 (0.011)	0.006 (0.010)	0.013 (0.006)
	TIII	0.040 (0.009)	0.011 (0.022)	0.030 (0.006)
PBTZ169 (25)	TI	-0.004 (0.014)	-0.003 (0.009)	-0.003 (0.006)
	TIII	-0.003 (0.007)	0.030 (0.003)	0.011 (0.004)
PBTZ169 (50)	TI	-0.013 (0.013)	0.035 (0.010)	0.014 (0.008)
	TIII	0.006 (0.010)	0.008 (0.015)	0.007 (0.003)
PBTZ169 (100)	TI	-0.003 (0.014)	0.020 (0.010)	0.010 (0.007)
	TIII	-0.004 (0.010)	0.031 (0.016)	0.013 (0.004)
OPC-167832 (1.25)	TI	0.041 (0.011)	-0.002 (0.010)	0.014 (0.008)
	TIII	0.028 (0.010)	0.003 (0.014)	0.017 (0.005)
OPC-167832 (5)	TI	0.040 (0.016)	0.023 (0.012)	0.030 (0.007)
	TIII	0.061 (0.006)	0.008 (0.019)	0.036 (0.007)
OPC-167832 (20)	TI	0.043 (0.009)	0.018 (0.011)	0.029 (0.006)
	TIII	0.066 (0.009)	0.005 (0.014)	0.040 (0.007)

C

Drug (mg/kg)	k_[0-28]	k_[28-56]	k_[0-56]
PreRx & Untreated	-0.010 (0.009)	-0.021 (0.009)	-0.016 (0.004)
TBA-7371 (50)	-0.022 (0.005)	-0.013 (0.006)	0.005 (0.004)
TBA-7371 (100)	0.035 (0.004)	0.021 (0.007)	0.028 (0.003)
TBA-7371 (200)	0.046 (0.006)	0.033 (0.007)	0.039 (0.003)
PBTZ169 (25)	-0.003 (0.008)	0.013 (0.010)	0.005 (0.004)
PBTZ169 (50)	-0.004 (0.007)	0.032 (0.008)	0.014 (0.004)
PBTZ169 (100)	-0.002 (0.009)	0.030 (0.010)	0.016 (0.004)
OPC-167832 (1.25)	0.044 (0.005)	0.023 (0.006)	0.033 (0.003)
OPC-167832 (5)	0.052 (0.005)	0.030 (0.006)	0.041 (0.003)
OPC-167832 (20)	0.071 (0.005)	0.012 (0.007)	0.042 (0.005)

Suppl. Table 2: *P* values using a two-way ANOVA with adjustments for multiple comparisons using one-way Tukey's test (pairwise). (**A**) *P* values for 4 weeks of treatment in lungs, (**B**) *P* values for 4 weeks of treatment in spleens, (**C**) *P* values for 8 weeks of treatment in lungs, (**D**) *P* values for 8 weeks of treatment in spleens.

**Suppl. Table 2A**: DprE1 inhibitor/Dose (frequency) 4 weeks lungs		TBA-7371			PBTZ169			OPC-167832		
		50 (BID)	100 (BID)	200 (BID)	25 (QD)	50 (QD)	100 (QD)	1.25 (QD)	5 (QD)	20 (QD)
TBA-7371	50 (BID)	NA	0.9843	0.8372	0.9980	0.8141	0.9726	0.5049	0.0576	0.0008
	100 (BID)		NA	0.9998	0.7197	0.2166	0.4828	0.9773	0.4471	0.0202
	200 (BID)			NA	0.3771	0.0658	0.1965	0.9998	0.7862	0.0825
PBTZ169	25 (QD)				NA	0.9952	>0.9999	0.1348	0.0070	<0.0001
	50 (QD)					NA	>0.9999	0.0150	0.0004	<0.0001
	100 (QD)						NA	0.0567	0.0022	<0.0001
OPC-167832	1.25 (QD)							NA	0.9735	0.2626
	5 (QD)								NA	0.8987
	20 (QD)									NA

**Suppl. Table 2B:** DprE1 inhibitor/Dose (frequency) 4 weeks spleens		TBA-7371			PBTZ169			OPC-167832		
		50 (BID)	100 (BID)	200 (BID)	25 (QD)	50 (QD)	100 (QD)	1.25 (QD)	5 (QD)	20 (QD)
TBA-7371	50 (BID)	NA	0.6775	0.0499	0.0356	0.0278	0.1827	0.0992	0.0050	<0.0001
	100 (BID)		NA	0.8914	<0.0001	<0.0001	0.0011	0.9671	0.4210	0.0004
	200 (BID)			NA	<0.0001	<0.0001	<0.0001	>0.9999	0.9968	0.0334
PBTZ169	25 (QD)				NA	>0.9999	0.9989	<0.0001	<0.0001	<0.0001
	50 (QD)					NA	0.9974	<0.0001	<0.0001	<0.0001
	100 (QD)						NA	<0.0001	<0.0001	<0.0001
OPC-167832	1.25 (QD)							NA	0.9771	0.0152
	5 (QD)								NA	0.2190
	20 (QD)									NA

**Suppl. Table 2C:** DprE1 inhibitor/Dose (frequency) 8 weeks lungs		TBA-7371			PBTZ169			OPC-167832		
		50 (BID)	100 (BID)	200 (BID)	25 (QD)	50 (QD)	100 (QD)	1.25 (QD)	5 (QD)	20 (QD)
TBA-7371	50 (BID)	NA	0.4507	0.9203	>0.9999	0.8839	0.8460	0.7032	0.0033	0.0009
	100 (BID)		NA	0.9944	0.2459	0.9989	0.9991	>0.9999	0.5087	0.2678
	200 (BID)			NA	0.7682	>0.9999	>0.9999	>0.9999	0.1050	0.0376
PBTZ169	25 (QD)				NA	0.7132	0.6473	0.4714	0.0008	0.0002
	50 (QD)					NA	>0.9999	>0.9999	0.1808	0.0737
	100 (QD)						NA	>0.9999	0.1619	0.0625
OPC-167832	1.25 (QD)							NA	0.2731	0.1181
	5 (QD)								NA	>0.9999
	20 (QD)									NA

**Suppl. Table 2D:** DprE1 inhibitor/Dose (frequency) 8 weeks spleens		TBA-7371			PBTZ169			OPC-167832		
		50 (BID)	100 (BID)	200 (BID)	25 (QD)	50 (QD)	100 (QD)	1.25 (QD)	5 (QD)	20 (QD)
TBA-7371	50 (BID)	NA	<0.0001	<0.0001	>0.9999	0.2477	0.0864	<0.0001	<0.0001	<0.0001
	100 (BID)		NA	0.1449	<0.0001	0.0116	0.0269	0.9074	0.0303	0.0145
	200 (BID)			NA	<0.0001	<0.0001	<0.0001	0.8892	0.9994	0.9929
PBTZ169	25 (QD)				NA	0.2307	0.0749	<0.0001	<0.0001	<0.0001
	50 (QD)					NA	>0.9999	0.0001	<0.0001	<0.0001
	100 (QD)						NA	0.0003	<0.0001	<0.0001
OPC-167832	1.25 (QD)							NA	0.5227	0.3591
	5 (QD)								NA	>0.9999
	20 (QD)									NA
